# Erosion of the Capital City Advantage in Child Survival and Reproductive, Maternal, Newborn, and Child Health Intervention Coverage in Sub-Saharan Africa

**DOI:** 10.1007/s11524-023-00820-0

**Published:** 2024-05-20

**Authors:** Agbessi Amouzou, Dessalegn Y. Melesse, Fernando C. Wehrmeister, Leonardo Z. Ferreira, Safia S. Jiwani, Sethson Kassegne, Abdoulaye Maïga, Cheikh M. Faye, Tome Ca, Ties Boerma, For The Countdown to 2030 Regional Collaboration in Sub-Saharan Africa

**Affiliations:** 1 Johns Hopkins Bloomberg School of Public Health, 615 N. Wolfe Street, Baltimore, MD 21205 USA; 2 Institute for Global Public Health, Department of Community Health Sciences, University of Manitoba, Winnipeg, MB Canada; 3 International Center for Equity in Health, Federal University of Pelotas, Pelotas, Brazil; 4 West African Health Organization, Bobo-Dioulasso, Burkina Faso; 5 African Population and Health Research Center, Dakar, Senegal

**Keywords:** Urban health, Capital cities, Child mortality, Intervention coverage, RMNCH, Place of residence

## Abstract

The place of residence is a major determinant of RMNCH outcomes, with rural areas often lagging in sub-Saharan Africa. This long-held pattern may be changing given differential progress across areas and increasing urbanization. We assessed inequalities in child mortality and RMNCH coverage across capital cities and other urban and rural areas. We analyzed mortality data from 163 DHS and MICS in 39 countries with the most recent survey conducted between 1990 and 2020 and RMNCH coverage data from 39 countries. We assessed inequality trends in neonatal and under-five mortality and in RMNCH coverage using multilevel linear regression models. Under-five mortality rates and RMNCH service coverage inequalities by place of residence have reduced substantially in sub-Saharan Africa, with rural areas experiencing faster progress than other areas. The absolute gap in child mortality between rural areas and capital cities and that between rural and other urban areas reduced respectively from 41 and 26 deaths per 1000 live births in 2000 to 23 and 15 by 2015. Capital cities are losing their primacy in child survival and RMNCH coverage over other urban areas and rural areas, especially in Eastern Africa where under-five mortality gap between capital cities and rural areas closed almost completely by 2015. While child mortality and RMNCH coverage inequalities are closing rapidly by place of residence, slower trends in capital cities and urban areas suggest gradual erosion of capital city and urban health advantage. Monitoring child mortality and RMNCH coverage trends in urban areas, especially among the urban poor, and addressing factors of within urban inequalities are urgently needed.

**Supplementary Information:**

The online version contains supplementary material available at 10.1007/s11524-023-00820-0.

## Introduction

Living in urban settings in low- and middle-income countries (LMICs) offers numerous health and economic advantages compared to rural areas. The urban areas are characterized by a concentration of health facilities, schools, employment opportunities, access to piped water and improved sanitation, and other infrastructures such as paved roads, electricity, access to media, and opportunities for better ways of living that promote health and development. While urban areas and cities have been targeted for socio-economic development, rural areas received less attention beyond dispersed rural development projects. Multiple factors have fueled massive rural-to-urban migration in most countries, creating rapid and often uncontrolled urbanization. The 2010 joint WHO and UN-Habitat report on hidden cities concluded that rapid urbanization will be among the most important global health issues of the twenty-first century [1, 2]. In 1990, just over one in four people in sub-Saharan Africa lived in urban areas. This proportion increased to 41% in 2020. Only six out of 51 countries in sub-Saharan Africa had half or more of their population living in urban areas in 1990, but by 2018 this number had grown to 19 countries. It is projected that close to half of the population in sub-Saharan Africa will be urban by 2030, and three in five by 2050 [3, 4].

Capital cities in sub-Saharan Africa are often the destination of mass rural–urban migration and concentrate a larger share of the urban population and infrastructure. Consequently, capital cities are expanding rapidly with crowded and growing slums or informal settlements as governments are unable to provide decent public housing or implement an effective urban growth and development plan [5, 6]. Such uncontrolled expansion presents serious threats to the health and living conditions of the urban poorest population and generates further health inequalities [1, 7]. These threats are further compounded by the high risk new migrants to cities face in adopting unhealthy lifestyles such as excessive alcohol consumption and tobacco use as well as increased exposure to environmental hazards [1]. The WHO and UN Habitat report highlighted the triple threats faced by city dwellers, including exposure to infectious diseases, non-communicable diseases and conditions, and injuries, including road traffic accidents and violence [1].

There is evidence that the rate of progress in increasing intervention coverage and reducing child mortality has slowed down in recent years in urban settings, including capital cities, compared to the rate of progress in rural areas [8]. This deceleration is occurring while health intervention coverage levels in urban areas are far from universal. Analysis of coverage of select interventions in 64 countries showed that progress in increasing national coverage rates were mainly due to increasing trends in rural areas [2, 9]. Similarly, Kimani-Murage and colleagues found in their assessment of childhood mortality trends in Kenya that the slower trends in urban areas were the result of poor living conditions in these areas, especially in slums. They concluded that the “urban advantage has seemingly been wiped out” [10, 11].

The long-held urban health advantage may be disappearing in many countries in sub-Saharan Africa. Analyses comparing urban to rural residence are generally featured in health equity analysis. However, except for a few recent studies that have looked at trends, these analyses are generally limited to cross-sectional assessments. Furthermore, capital or large cities are rarely separated out and compared to other urban and rural areas. Global analysis and publications by the UN-Habitat constitute key advances in the understanding of urban and city demographics, development, and quality of life of urban dwellers, but have not systematically focused on trends in outcomes such as health intervention coverage and child mortality [12–14].

This paper analyzes patterns of inequalities in intervention coverage and neonatal and under-five mortality in sub-Saharan Africa by comparing trends in capital cities to those in other urban and rural areas. It also compares rates of progress between the urban poor with both urban non-poor and rural populations. It presents evidence of rapidly eroding urban advantage in many capital cities and rural populations catching up with urban populations. The slowdown of progress in urban areas is marked by a large health intervention coverage gap between the urban poor and the urban rich. Women and children living in the poorest households in urban area have no health advantage over rural women and children in terms of health intervention coverage.

## Data and Methods

We used data from Demographic and Health Surveys (DHS) and Multiple Indicator Cluster Surveys (MICS). The mortality analysis includes 163 surveys in 39 countries with the latest survey conducted between 2010 and 2020. All surveys were carried out between 1990 and 2020 with a median of 2007. The average number of surveys per country is five, with 77% of the countries with four or more surveys.

The coverage of RMNCH was assessed through the composite coverage index (CCI) [15]. A total of 39 countries with 141 surveys spanning from 1993 to 2021 were included in the coverage analysis. The median year across surveys was 2011. Thirty-five of 39 countries have data for at least two surveys, with an average of 4.4 surveys per country.

We grouped the countries into four sub-regions, based on the United Nations Population Division regional grouping: West, Central, Eastern, and Southern Africa (see the list in supplementary information Table 1). Additional data on trends in urbanization and population size of urban cities were retrieved from the 2018 World Urbanization Prospects produced by United Nations Population Division [3].

### Neonatal and Under-Five Mortality Rate

We computed under-five mortality rate (U5MR) using the full birth history module included in each survey. For each woman of reproductive age interviewed during the survey, the module collects detailed information on all her live births, their survival status, age and date of birth, and age at death for deceased children. We computed under-five mortality rates for the 5-year periods before the survey by place residence, assuming that the place of residence at the time of the survey did not change in the last 5 years. We also computed neonatal mortality rate by place of residence for the most recent survey. For trends assessment, we limited the analysis to the 38 countries with mortality data from at least two surveys (excluding DHS 2015 Angola). The mortality rate is computed using a synthetic cohort life table approach [16].

### RMNCH Coverage Measure

The CCI is a summary measure of selected coverage interventions along the RMNCH continuum of care, including reproductive health (proportion of women with a demand for family planning satisfied with modern methods), maternal health (at least four antenatal care visits and skilled birth attendant), child immunization (measles, DPT3, and BCG vaccinations), and child illness treatment indicators (oral rehydration salts for episodes of diarrhea and care-seeking for symptoms of acute respiratory infections) [17–19]. The CCI has been shown to correlate well with coverage of individual interventions and health status measures such as mortality and nutrition status [15]. The CCI is affected by small sample sizes, especially for child illness treatment and immunization indicators, making disaggregation by other characteristics of interest challenging. For this analysis, the CCI has been slightly modified to address the challenge with small sample sizes resulting from these indicators and the disaggregation by place of residence. Specifically, the child illness indicators were replaced with a combined indicator on care-seeking for childhood diarrhea or symptoms of suspected pneumonia in the 2 weeks preceding the survey. All coverage indicators included in the CCI calculation were measured at the time of the surveys. The maternal health indicators were based on births in the past 2 years preceding the survey.

### Place of Residence

Residence information is collected during each survey and distinguishes urban area from rural, based on country-specific definition. Additionally, the capital/largest city or region is also reported. Our residence variable includes three categories (capital/largest city, other urban and rural areas) and corresponds to the place of residence of respondent women at the time of the survey interviews. In Nigeria and Tanzania, we used the largest city (Lagos and Dar es Salaam, respectively) instead of the much smaller capital city. For the few surveys in which the capital city was not recorded, we used the urban area in the region within which the capital city is located.

### Statistical Analysis

We first compared neonatal and under-five mortality and CCI by place of residence to assess country patterns. This analysis helped identify countries where urban areas were not advantageous for the health outcomes. We then fit a multilevel linear regression of under-five mortality on year in the entire data set and for each specific sub-region to derive average trends in each area of residence. We decided on the choice of a linear regression after assessing trends in mortality using non-parametric polynomial regression, which indicated a linear trend in the data, except in the Southern region. The multilevel regression model used the country as first level and the survey as second level and thus takes into account specific country trends. We limited the trend assessment to the period 2000–2015. Data points were located in the middle of the 5-year recall period (2.5 years before the year of survey). The analysis was not weighted by country population since we were not seeking to generate population representative estimates. Predicted trends for all countries and for each sub-region are used to estimate the absolute mortality gaps between capital cities and other urban areas and between other urban and rural areas for the year 2000, 2005, 2010, and 2015. We explored inequity in neonatal and under-five mortality by wealth quintiles focusing on capital city residents. Due to small sample sizes, we grouped countries according to whether the capital city had higher or lower mortality compared to the rural area. For U5MR, we categorized countries into groups 1A and 2A. Group 1A includes countries where capital city U5MR is higher than that of rural areas and group 2A the rest of the countries. We used a similar grouping for NMR and grouped countries into group 1B (countries where NMR is higher in capital cities compared to rural areas) and group 2B (the rest of the countries).

Similar multilevel regression analysis was carried out for coverage, with two levels (country and survey) to estimate average trends in coverage in each area of residence for all countries and by specific sub-region. We estimated predicted absolute coverage gap between capital cities and other urban and rural areas for years 2000, 2005, 2010, and 2015. Finally, we described coverage among poorest urban, defined as urban dwellers that are in the bottom 20% of national wealth quintiles and compared them to richest urban who were those dwellers in the top 20% national wealth quintile. This country-specific analysis was not implemented for mortality given small sample sizes. We compared coverage rates among urban poor to urban richest and rural populations. This analysis used only the most recent available survey in each country.

All analyses were performed with the STATA statistical software and some graphs generated in Microsoft Excel.

## Results

Sub-Saharan Africa is urbanizing rapidly. The proportion of the population living in urban settings increased by 10 percentage points between 2000 and 2020, from 31 to 41% [3]. Within the region, the level of urbanization varies largely across the sub-regions and countries (supplementary information Table 2 and Figs. 8 and 9). Southern Africa is the most urbanized with over half (57%) of its population living in urban area, driven mostly by Botswana (69%), South Africa (66%), and Namibia (50%). The least urbanized sub-region is East Africa with 28% urban population, followed by West Africa (46%) and Central Africa (50%). The two most populous countries in the region are not the most urbanized: urbanization rate is 50% and 21% in Nigeria and Ethiopia, respectively. Urban primacy, where the population in the capital city is at least twice as high as the second largest city, is observed in almost all countries. About 30 countries have capital cities with over one million population. Lagos in Nigeria and Kinshasa in the Democratic Republic of Congo are by far the largest capital cities with over 13 million population, followed by Luanda in Angola, Dar Es Salaam in Tanzania, and Johannesburg in South Africa with population over 5 million.

### Neonatal and Under-Five Mortality by Residence

Across the countries included in the analysis, the median U5MR derived from latest surveys is highest in rural areas (69.0 per 1000 live births). Capital cities and other urban have similar median (59.5 and 61.7 per 1000 live births, respectively) (Supplementary information Fig. 10 and Table 3). Similar typical mortality patterns by place of residence are observed in Central and West Africa, with lowest median rate in capital cities and highest in rural areas. East Africa and Southern Africa showed a different pattern with U5MR lowest in other urban and almost similar in capital cities and rural areas. The interquartile range is wider in Central and West regions, indicating greater variability compared to the Eastern and Southern regions.

The gap in neonatal mortality by place of residence (Supplementary information Fig. 11 and Table 4) shows a slightly different pattern with smaller gaps. The median across all countries is similar for the three areas (26 deaths per 1000). Besides West Africa, where the median neonatal mortality rate is lower in capital cities than in other urban or rural (21 versus 29 per 1000), the gap in the median is small or inexistant in the other three sub-regions.

**Fig. 1:**
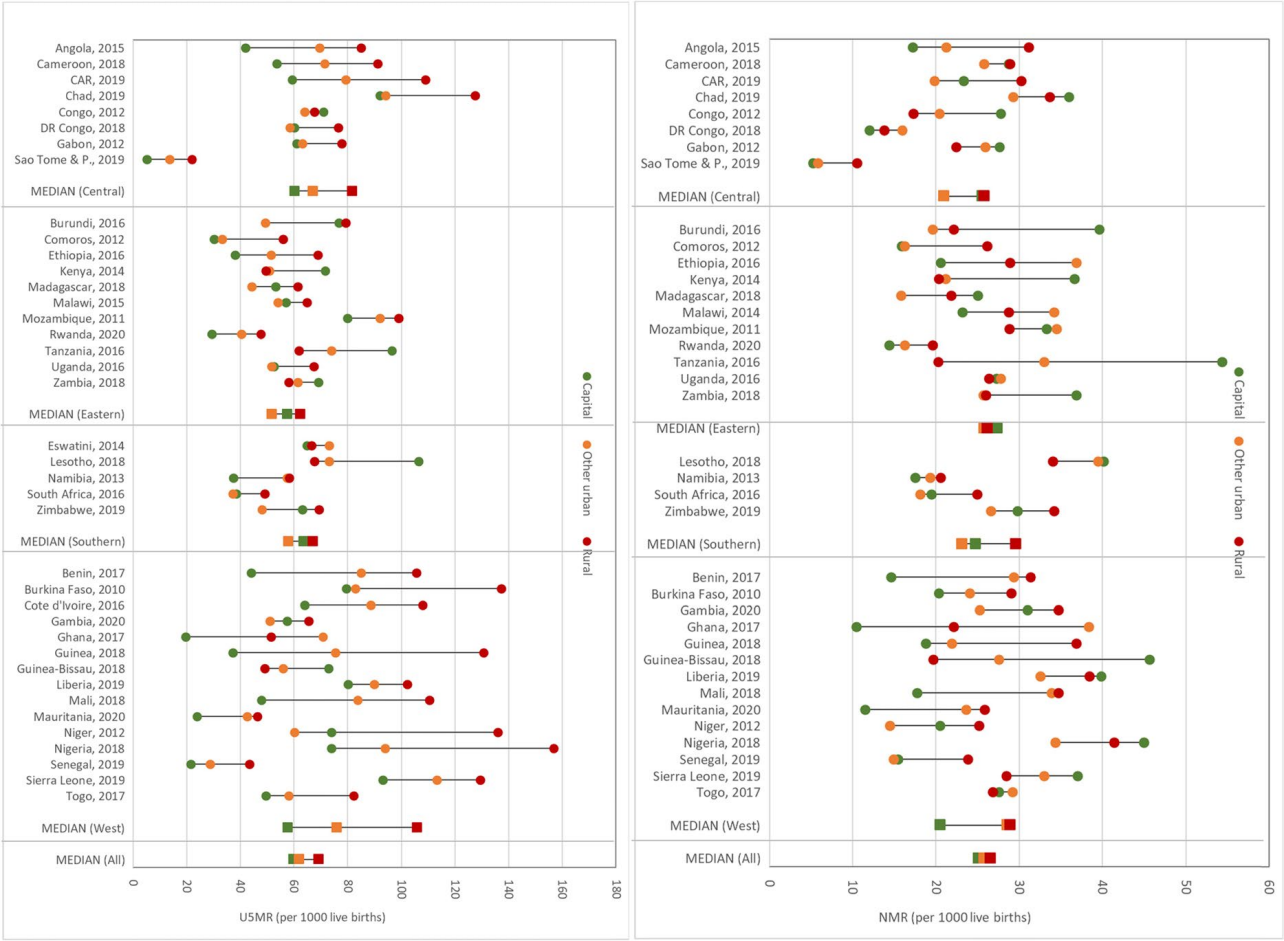
Neonatal and under-five mortality rates by place of residence and country (latest DHS or MICS survey, 5-year rates)

Figure 1 shows under-five and neonatal mortality by place of residence for specific countries included in the analysis, using the latest surveys. In general, the rural area is the place with highest U5MR. However, several capital cities such as Bujumbura in Burundi, Maseru in Lesotho, Dar es Salaam in Tanzania, Nairobi in Kenya, Lusaka in Zambia, and Bissau in Guinea-Bissau had higher or similar level of under-five mortality as the rural areas. Congo and Eswatini had a small gap in U5MR by place of residence, although their mortality level is still high (above 60 deaths per 1000). For eight countries, the capital city does not show any advantage over other urban areas, although the rural areas remain the worse place for children under-five. These include Chad, DR Congo, Gabon, Comoros, Malawi, Uganda, South Africa, and Burkina Faso. U5MR gaps by place of residence were widest in West Africa and smallest in Eastern Africa. The patterns of the gap in NMR by place of residence are more irregular than those of U5MR as these rates are more affected by smaller sample sizes. Many capital cities in all four sub-regions showed higher or similar NMR level as the rural areas. In the central sub-region, N’Djamena in Chad, Brazzaville in Congo, and Libreville in Gabon had the highest NMR. In the Eastern sub-region, these included Bujumbura in Burundi, Nairobi in Kenya, Maputo in Mozambique, Dar Es Salaam in Tanzania, and Lusaka in Zambia. Of the four capital cities included in the Southern sub-region, Maseru in Lesotho had the highest. In West Africa, Bissau in Guinea-Bissau, Monrovia in Liberia, Lagos in Nigeria, and Freetown in Sierra Leone were the capital cities where newborns had higher mortality than rural areas.

Overall trends in U5MR by place of residence over the past two decades in sub-Saharan Africa show rapid decline in all three places of residence, but with narrowing of the gaps due primarily to faster decline in rural areas and slower decline in capital cities, allowing rural areas to catch up quickly with other areas (see supplementary information Fig. 12). The average annual rate of decline in U5MR was 4.1 (95% CI − 4.6, − 3.7) deaths per 1000 live births in rural areas compared to 3.4 (95% CI − 3.8, − 3.0) and 2.9 (95% CI − 3.4, − 2.5) deaths in other urban and capital cities, respectively. These trends contributed to closing the mortality gap between other urban and capital cities from 15 deaths per 1000 live births in 2000 to 7 in 2015, and the gap between rural areas and capital cities from 41 to 23 over the same period (Supplementary information Table 6).

**Fig. 2:**
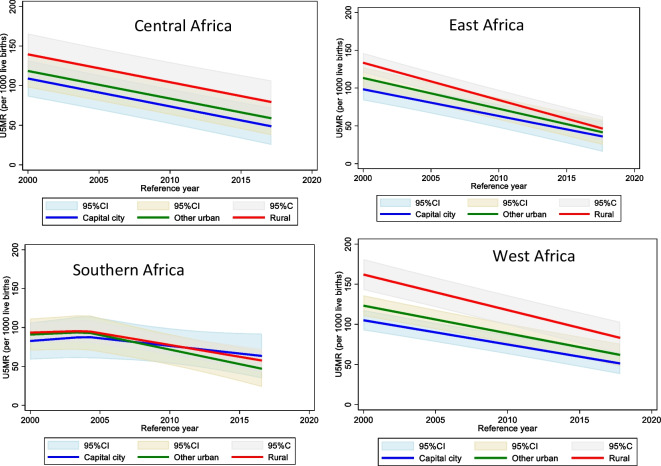
Trends in under-five mortality by residence and sub-region in sub-Saharan Africa

Figure 2 shows predicted trend by region with 95% confidence intervals for capital cities, other urban areas, and rural areas. Tables 5, 6, and 7 in the supplementary information show the slopes, the mortality gaps, and the predicted mortality estimates, respectively. In Central Africa, U5MR declined at the same pace in rural and capital cities. By 2015, capital cities had lower U5MR than other urban areas, which in turn has lower mortality than rural area. In Eastern Africa, the predicted large mortality gap between the three places of residence in 2000 closed almost completely by 2015. The gap between rural and capital cities in 2000, estimated at 35 deaths per 1000 live births, closed to 14 deaths per 1000 live birth by 2015. The gap between other urban and capital cities halved from 15 to 7, while the rural-other urban gap reduced from 20 to 7. West Africa also showed a narrowing of the gaps although a substantial gap capital city—rural areas remained by 2015. The gap between capital cities and rural areas reduced substantially from 57 to 36 deaths per 1000. In Southern Africa, average mortality increased in all three areas until around 2005 before it started to level off. Mortality differences between the three areas were small, with other urban area showing a consistent advantage and capital cities losing their initial advantage.

**Fig. 3:**
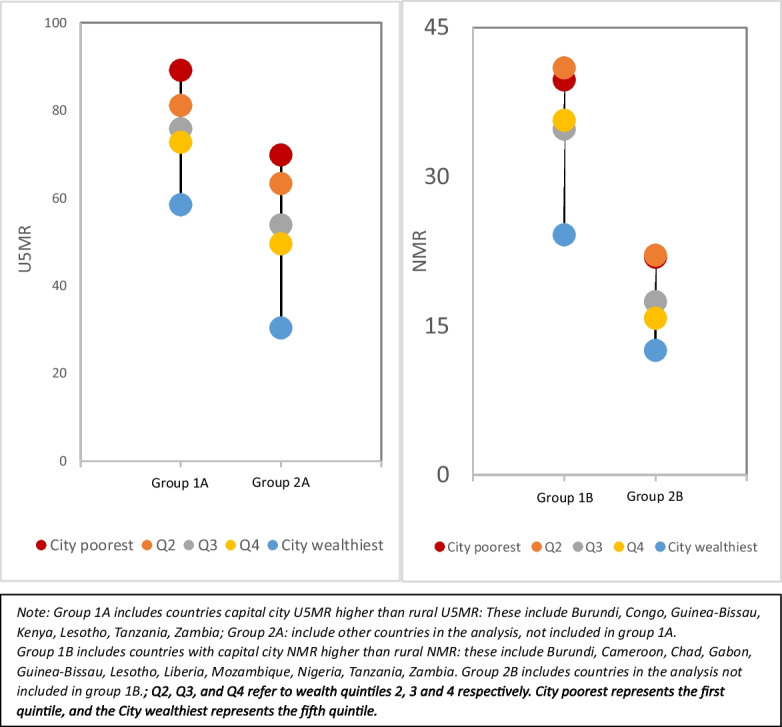
Under-five and neonatal mortality by wealth quintile comparing the group of countries with highest mortality in the capital city to others

Figure 3 shows pooled U5MR and NMR by wealth quintiles within capital cities. Countries were pooled according whether capital city U5MR is higher than rural U5MR (group 1A versus group 2A) or capital city NMR is higher than rural NMR (group 1B versus group 2B). With regard to U5MR, group 1A has higher mortality than group 1B but the inequality patterns by wealth quintile is similar between the two, with a slightly wider gap for group 2A. Both groups show top inequality, with the wealthiest quintile (often referred to as top quintile) having a substantially lower mortality than the bottom four quintiles. However, the wealthiest in group 1A fare much worse than wealthiest in group 2A, with mortality rate comparable to the bottom two and three quintiles of group 2A. For NMR, group 1B also shows higher NMR than group 2B. However, the inequality patterns are different. Group 1B shows top inequality with a much wider top–bottom gap, while group 2B has a linear inequality. A prominent feature is that the wealthiest in group 1B appears worse than the poorest in group 2B. Thus, in capital cities with higher mortality than rural areas, not only mortality is highest among the poorest groups but the wealthiest groups do not also achieve their full survival potentials.

### Coverage of RMNCH Interventions by Residence

**Fig. 4:**
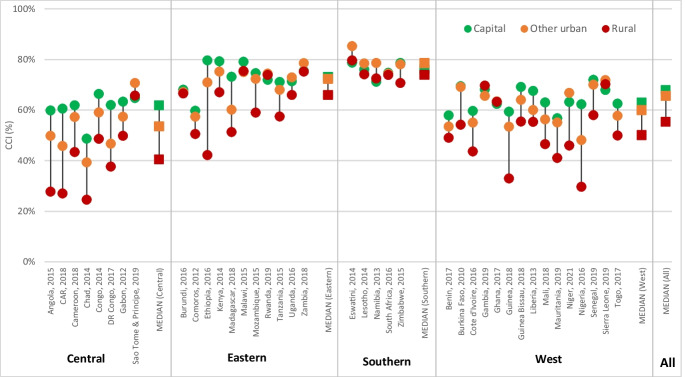
Composite coverage index by place of residence by country in sub-Saharan Africa (latest DHS/MICS survey), ordered by sub-region and coverage level

Figure 4 presents the CCI by place of residence, country, and sub-region, based on latest available survey. Figure 13 and Table 8 in the supplementary information show the median coverage values by sub-region. Overall, the coverage levels and their distribution show an expected pattern by place of residence: highest in capital cities with a median of 68%, and in other urban areas (median = 66%) and lowest in rural areas (median 55%). A few countries showed no coverage gap by place of residence. These included Burundi, Rwanda, Malawi, Zambia, Lesotho, South Africa, Gambia, Ghana, and Sierra Leone. Coverage gaps are smallest in countries with high coverage levels located in Southern and Eastern regions. The coverage gaps between capital city and other urban are generally small.

**Fig. 5:**
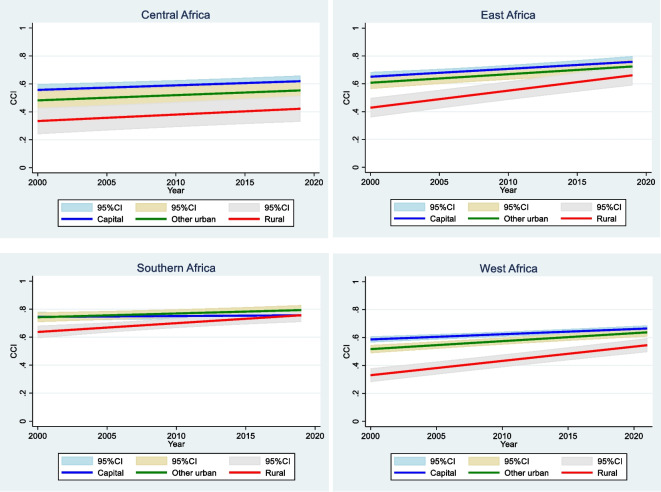
Predicted trends in composite coverage index (CCI) by sub-region in sub-Sahara Africa

Coverage levels have increased steadily in each place of residence during the past two decades, with a narrowing of the gaps by 2015, due to rural areas progressing faster (Fig. 11 and Table 9 in the supplementary information). The average CCI increased from 61 to 68% between 2000 and 2015 in capital cities (Table 10 in the supplementary information). It went from 56 to 64% in other urban and from 39 to 54% in rural areas. The predicted gap in capital cities-rural areas closed by 8 percentage points: from 22 percentage points to 14 over this period. The gap in other urban–rural areas went from 17 percentage points to 10 and that of capital cities-other urban from 5 to 4 percentage points (Table 11 in the supplementary information). The breakdown of the trends analysis by sub-region shows similar trend patterns in each sub-region with the rural areas catching up quickly with the capital cities and other urban, particularly in East, Southern, and West Africa (Fig. 5).

### Coverage among the Urban Poor

**Fig. 6:**
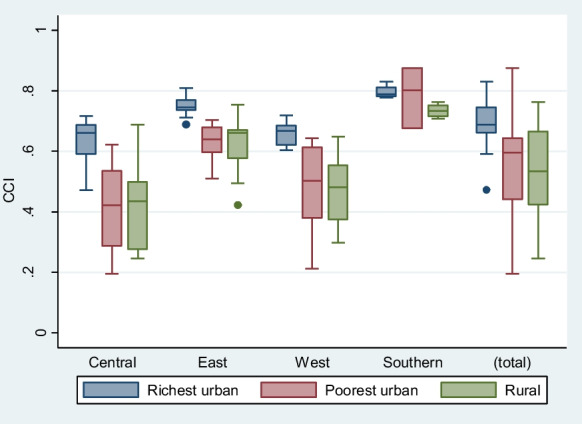
Median and interquartile range for the composite coverage index (CCI) for richest urban, poorest urban and rural by sub-region in sub-Saharan Africa

**Fig. 7:**
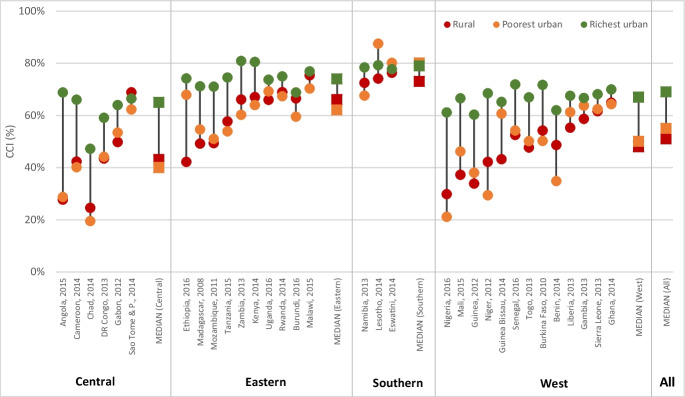
Composite coverage index for richest urban, poorest urban, and rural population by country

Figure 15 and Table 11 in the supplementary information show the medians CCI and interquartile ranges for the poorest and richest urban and rural population by sub-region and for all the countries. Overall, the median across all countries is highest among the richest urban (69%) and lowest among the rural populations (51%), with the poorest urban in between (55%). However, the distribution of the CCI among the poorest urban is much wider than for the other two groups. The breakdown by sub-regions reveals a different pattern (Fig. 6). Except for the Southern sub-region, coverage level among the urban poor is similar to that of rural areas in all sub-regions. This result is confirmed by the predicted regression line fitted separately for urban rich, urban poor, and the rural populations, which shows overlapping trends for the urban poor and the rural (Fig. 16 in the supplementary information). There is however a large gap between the richest urban and the poorest urban in all these three sub-regions, ranging from 25 percentage points in Central African to 12 in East Africa. This result is further reinforced in the country-specific analysis (Fig. 7). In all countries in Central and East Africa, the poorest urban has similar or lower coverage rate as the rural population. In West Africa, the pattern is mixed although the coverage rates among the poorest urban and rural populations appear generally close. Similarly, there is a large coverage gap between the poorest urban and the richest urban, particularly in Angola, Nigeria, Niger, Chad, Benin, and Cameroon where the gap is over 25 percentage points (Fig. 7).

## Discussion

Low-income countries, especially those in the sub-Saharan African region, are urbanizing rapidly, fueled by the rural-to-urban migration. While 41% of the total population in Sub-Saharan Africa lived in urban areas in 2020, it is projected that by 2050, close to 60% of the region’s population will be urban area [3]. The rapid urbanization within a context of chronic deficiency in urban planning leads to the growth and uncontrolled expansion of slum populations, with greater health risks for the urban population, especially for children and women [5, 6, 20]. In general, studies have shown that urban areas have better child health outcomes than rural areas, due mainly to better socio-economic conditions and health infrastructure in urban areas [2, 21]. Several studies, however, have demonstrated the poorer condition of the urban poor children and women compared to rural children with regards to mortality and malnutrition [22–27].

There are major inequalities in neonatal and child mortality and coverage of RMNCH interventions between capital cities and other urban and rural areas in many of sub-Saharan African countries. Our analysis highlights the condition of urban poor, in comparison to the rural population and urban rich population. While under-five mortality rate is lowest in capital cities compared to other areas in the overall sub-Saharan African countries included in the analysis, important and new evidence emerged from the analysis at sub-region and country level.

First, in all sub-regions, child mortality gaps by place of residence are closing rapidly due mainly to rural areas catching up quickly with other urban areas and capital cities with faster trends in mortality reduction, especially in East Africa. It can be argued that the faster decline in rural areas compared to capital cities or other urban areas is due to high baseline mortality in rural areas in the 1990s. However, mortality levels in capital cities or other urban areas were not particularly low either. The median U5MR based on latest surveys was 57 per 1000 live births in capital cities, 64 in other urban, and 78 in rural areas. In addition, multiple studies have shown the disappearance of urban or city advantage, following a slower mortality decline due to  persistent high mortality in capital cities [10, 25, 28].

Second, the average child mortality gap by place of residence appears to have almost closed by 2015 in East Africa and reduced substantially in West Africa. Countries such as Kenya, Tanzania, Zambia, and Burundi in Eastern Africa, Guinea-Bissau in West Africa, and Lesotho in Southern Africa showed higher mortality in capital cities than in other urban or rural areas. Gaps in neonatal mortality are more irregular with many capital cities across all sub-Saharan African regions experiencing similar or higher level of neonatal mortality than rural or other urban areas. Smaller sample sizes may have caused the irregular patterns observed for neonatal mortality but the higher mortality in capital cities, consistent with mortality among children under-five is suggestive of eroding capital city survival advantage over other urban and rural areas. While the effects of HIV/AIDS have been raised in the past as the factor of higher child mortality in urban area than rural in countries particularly affected by the epidemic, it seems that this factor is no longer the only contributing factor to the observed results [29–31]. Furthermore, studies in city slums have shown that slum residents have higher burden of infectious diseases, including HIV and higher mortality burden [32]. There are pervasive inequities by wealth in capital cities, with the top quintile faring way better than the remaining 80% of the city dwellers. We found that, in countries where U5MR or NMR is higher in capital cities compared to other urban or rural areas, not only overall mortality is higher in these cities, but the wealthiest group in these cities has much higher mortality compared to the wealthiest in other capital cities where mortality is lower. Thus, addressing mortality inequities by targeting the poorest groups will be advantageous to all groups. Indeed, one of the conclusion of the joint WHO and UN-Habitat’s 2010 report on hidden African cities was the fact that health inequities are detrimental to all city dwellers [1].

Third, analysis using the composite coverage index (CCI) of RMNCH did not mirror the results observed for child mortality. Coverage of essential health services in most capital cities and urban areas was higher than rural coverage in almost all countries. Although the gaps have reduced by place of residence, the capital city advantage over other areas has remained over time in all sub-regions, except in Southern Africa. There were few countries such as Ghana, Lesotho, Burundi, Malawi, Namibia, Sierra Leone, and Zambia where there were no coverage differentials by place of residence and the coverage level was above 60% in each of these countries. Although the coverage patterns by place of residence differed from that of the U5MR, the comparison of the poorest urban population to the rural population and the richest urban population provided important findings. In Central, Eastern, and West sub-regions, the coverage levels in the poorest urban population were similar to those observed in rural areas and far below that of the richest population. The city poor therefore do appear to show greater health advantage than the rural population.

This study has several limitations. The definition of place of residence evolved overtime within countries and may not be comparable across countries and time. Urban areas also expand due to redefinition of boundaries or reclassification of rural areas. We have used the definition as included in the existing datasets, which is consistent with countries’ definition but did not account for possible changes over time. The country-specific descriptive inequality analysis did not take into account standard errors of the mortality and coverage level estimates. It is therefore possible that some of the gaps shown may not be statistically significant, although the consistency in the trends observed suggested that they were programmatically relevant. Furthermore, sub-regional level analysis combining estimates from multiple countries improved the power of the analysis. The sub-regional analysis included only countries with available data, which may not be fully representative of the sub-region. The Southern region has been particularly affected by this limitation. The analysis was not weighted by country population, and therefore, sub-regional estimates should not be taken as population-representative estimates at the sub-region level. The extent to which surveys are able to identify the urban poor, especially those living in slums, may also be compromised and affect the results. Furthermore, the mortality experience of recent urban residents may refer to the time they were living in rural areas. We did not take into account the duration of stay in the urban area at the time of the survey or the place of death. We have limited the mortality estimates to the 5 years preceding the survey to reduce the effect of this limitation. The individual women data included in the analysis did not exclude women visitors in the households captured during the household interview. We expect these to be negligible and will not alter the findings.

There are several implications stemming from the results. First, inequality assessments must go beyond the traditional urban–rural disaggregation to incorporate within urban inequalities, including capital cities and the slum population. This can easily be realized if data collection initiatives include enough sample in urban areas and specifically oversample slum populations. Although most DHS and MICS surveys include categories for capital cities or region, rarely the sampling is designed to include sufficient representation of the slum population. We acknowledge that slum areas, especially those that are growing fast, are hard to sample because of poor sampling frames and unorganized settlements. Geospatial approaches in which slum areas are identified and sampled and linked to data on health services may offer a more actionable agenda for targeting of interventions, sometimes referred to as precision public health [33, 34]. Second, as countries strive to achieve universal health coverage, it is essential to understand the underlying reason of the poor outcomes in slum populations and the slowing down of progress in cities. While cities often provide greater opportunities for accessing healthcare, education, and employment, the urban poor or the slum population may be shut out of these opportunities. Other studies have suggested that unobservable factors that determined the choice of slum residence may also determine care seeking. Therefore, improving socio-economic and living conditions of the slum population will result in better outcomes than building more health facilities [35]. Further research might seek to understand why the mortality patterns by place of residence are not consistent with coverage patterns and to disentangle separate differential effects of quality of health care versus urbanization or growth of the slum population. Finally, while redressing health strategies for more attention to urban poor and city slums, it is essential that global and country efforts to address universal health coverage maintain priority in rural areas where most people live in low-income countries, and which remain most vulnerable to environmental health hazards and health access and utilization obstacles.

## Supplementary Information

Below is the link to the electronic supplementary material.

Supplementary file1 (DOCX 132 KB)

## Data Availability

Data used in this study are publicly available on the Demographic and Health Survey website (dhsprogram.com) and the Multiple Indicator Cluster Survey website (data.unicef.org). Reanalyzed data are available from the corresponding authors.
