# Supplementary material for: Erosion of the Capital City Advantage in Child Survival and Reproductive, Maternal, Newborn, and Child Health Intervention Coverage in Sub-Saharan Africa
Source: J Urban Health. 2024 May 20;101(Suppl 1):18–30. doi: 10.1007/s11524-023-00820-0 (PMC11602891; doi:10.1007/s11524-023-00820-0)
Supplement: Supplementary file 1 — Supplementary file1 (DOCX 132 KB) [file 11524_2023_820_MOESM1_ESM.docx]

**Supplementary Information**

**Table 1** Sub-Saharan country classification by sub-region

| **Central Africa (9)** | **East Africa (15)** | **Southern Africa (6)** | **West Africa (16)** |
| --- | --- | --- | --- |
| Angola, Cameroon, Central African Republic, Chad, Congo, Democratic Republic of the Congo, Equatorial Guinea, Gabon Sao Tome and Principe | Burundi Comoros Djibouti Eritrea Ethiopia Kenya Madagascar Malawi Mozambique Rwanda Somalia South Sudan Tanzania Uganda Zambia | Botswana Lesotho Namibia South Africa Swaziland Zimbabwe | Benin Burkina Faso Cape Verde Cote d'Ivoire The Gambia Ghana Guinea Guinea‐Bissau Liberia Mali Mauritania Niger Nigeria Senegal Sierra Leone Togo |

**Fig. 8** Proportion of urban population by region in sub-Saharan Africa

Note: the red dot is the percent urban in each country in the region, and the bars represent the proportion in each region

Source: Analysis of data from United Nations, Department of Economic and Social Affairs, Population Division (2018). World Urbanization Prospects: The 2018 Revision, custom data acquired via website.

**Fig.9** Distribution of country population by place of residence in 2018

**Eastern Africa**

**Central Africa**

**Southern**

**Africa**

Source: Analysis of data from United Nations, Department of Economic and Social Affairs, Population Division (2018). World Urbanization Prospects: The 2018 Revision, custom data acquired via website.

**Table 2** Distribution of country population by place of residence (2018)and percent of urban population living slum (2014)

| **Region and country** | **Included in analysis** | **Capital city / largest city** | **Urban Pop (thousands)** | **Rural Pop (thousands)** | **Total Pop (thousands)** | **Capital city (thousands)** | **% Population in Capital city** | **% Population in other urban** | **% Population Rural** |
| --- | --- | --- | --- | --- | --- | --- | --- | --- | --- |
| **Central Africa** | | | | | | | | | |
| Angola | N | Luanda | 20 162 | 10 613 | 30 774 | 7 774 | 25 | 40 | 34 |
| Cameroon | Y | Yaoundé | 13 912 | 10 766 | 24 678 | 3 656 | 15 | 42 | 44 |
| Central African Rep. | Y | Bangui | 1 960 | 2 778 | 4 737 | 851 | 18 | 23 | 59 |
| Chad | Y | N'Djaména | 3 540 | 11 813 | 15 353 | 1 323 | 9 | 14 | 77 |
| Congo | Y | Brazzaville | 3 613 | 1 786 | 5 400 | 2 230 | 41 | 26 | 33 |
| Congo DR | Y | Kinshasa | 37 349 | 46 656 | 84 005 | 13 171 | 16 | 29 | 56 |
| Equatorial Guinea | N | Malabo | 948 | 366 | 1 314 | 297 | 23 | 50 | 28 |
| Gabon | Y | Libreville | 1 848 | 220 | 2 068 | 813 | 39 | 50 | 11 |
| Sao Tome and Pr. | Y | São Tomé | 152 | 57 | 209 | 80 | 38 | 34 | 27 |
| **TOTAL (Central Africa)** | | | **83 484** | **85 055** | **168 538** | **30 195** | **18** | **32** | **50** |
| **Eastern Africa** | | | | | | | | | |
| Burundi | N | Bujumbura | 1 462 | 9 755 | 11 216 | 899 | 8 | 5 | 87 |
| Comoros | Y | Moroni | 241 | 591 | 832 | 62 | 7 | 21 | 71 |
| Djibouti | N | Djibouti | 756 | 216 | 971 | 562 | 58 | 20 | 22 |
| Eritrea | N | Asmara | 2 079 | 3 109 | 5 188 | 896 | 17 | 23 | 60 |
| Ethiopia | Y | Addis Ababa | 22 328 | 85 207 | 107 535 | 4 400 | 4 | 17 | 79 |
| Kenya | Y | Nairobi | 13 772 | 37 179 | 50 951 | 4 386 | 9 | 18 | 73 |
| Madagascar | Y | Antananarivo | 9 767 | 16 496 | 26 263 | 3 058 | 12 | 26 | 63 |
| Malawi | Y | Lilongwe | 3 246 | 15 919 | 19 165 | 1 030 | 5 | 12 | 83 |
| Mauritius | N | Port Louis | 517 | 751 | 1 268 | 149 | 12 | 29 | 59 |
| Mayotte | N | Mamoudzou | 120 | 140 | 260 | 6 | 2 | 44 | 54 |
| Mozambique | Y | Maputo | 10 987 | 19 542 | 30 529 | 1 102 | 4 | 32 | 64 |
| Réunion | N | Saint-Denis | 879 | 4 | 883 | 147 | 17 | 83 | 0 |
| Rwanda | Y | Kigali | 2 152 | 10 350 | 12 501 | 1 058 | 8 | 9 | 83 |
| Seychelles | N | Victoria | 54 | 41 | 95 | 28 | 29 | 27 | 43 |
| Somalia | N | Mogadishu | 6 827 | 8 355 | 15 182 | 2 082 | 14 | 31 | 55 |
| South Sudan | N | Juba | 2 534 | 10 385 | 12 919 | 369 | 3 | 17 | 80 |
| Uganda | Y | Kampala | 10 525 | 33 745 | 44 271 | 2 986 | 7 | 17 | 76 |
| U. Rep. Tanzania | Y | Dar es Salaam | 19 959 | 39 133 | 59 091 | 6 048 | 10 | 24 | 66 |
| Zambia | Y | Lusaka | 7 664 | 9 946 | 17 609 | 2 524 | 14 | 29 | 56 |
| Zimbabwe | Y | Harare | 5 448 | 11 466 | 16 913 | 1 515 | 9 | 23 | 68 |
| **TOTAL (Eastern Africa)** | | | **121 316** | **312 328** | **433 643** | **33 307** | **8** | **20** | **72** |
| **Southern Africa** | | | | | | | | | |
| Botswana | N | Gaborone | 1 620 | 713 | 2 333 | 269 | 12 | 58 | 31 |
| Lesotho | Y | Maseru | 637 | 1 626 | 2 263 | 202 | 9 | 19 | 72 |
| Namibia | Y | Windhoek | 1 295 | 1 293 | 2 588 | 404 | 16 | 34 | 50 |
| South Africa | Y | Johannesburg | 38 087 | 19 312 | 57 398 | 5 486 | 10 | 57 | 34 |
| Eswatini | Y | Mbabane | 331 | 1 060 | 1 391 | 68 | 5 | 19 | 76 |
| **TOTAL (Southern Africa)** | |  | **41 970** | **65 974** | **6 429** | **10** | **54** | **36** | **36.05** |
| **West Africa** | | | | | | | | | |
| Benin | Y | Cotonou | 5 434 | 6 052 | 11 486 | 685 | 6 | 41 | 53 |
| Burkina Faso | Y | Ouagadougou | 5 799 | 13 953 | 19 752 | 2 531 | 13 | 17 | 71 |
| Cabo Verde | N | Praia | 364 | 190 | 553 | 168 | 30 | 35 | 34 |
| Côte d'Ivoire | Y | Abidjan | 12 647 | 12 259 | 24 906 | 4 921 | 20 | 31 | 49 |
| Gambia | Y | Banjul | 1 326 | 838 | 2 164 | 437 | 20 | 41 | 39 |
| Ghana | Y | Accra | 16 517 | 12 946 | 29 464 | 2 439 | 8 | 48 | 44 |
| Guinea | Y | Conakry | 4 717 | 8 335 | 13 053 | 1 843 | 14 | 22 | 64 |
| Guinea-Bissau | Y | Bissau | 827 | 1 080 | 1 907 | 558 | 29 | 14 | 57 |
| Liberia | Y | Monrovia | 2 483 | 2 371 | 4 854 | 1 418 | 29 | 22 | 49 |
| Mali | Y | Bamako | 8 093 | 11 014 | 19 108 | 2 447 | 13 | 30 | 58 |
| Mauritania | Y | Nouakchott | 2 437 | 2 103 | 4 540 | 1 205 | 27 | 27 | 46 |
| Niger | Y | Niamey | 3 665 | 18 647 | 22 311 | 1 214 | 5 | 11 | 84 |
| Nigeria | Y | Lagos | 98 611 | 97 264 | 195 875 | 13 463 | 7 | 43 | 50 |
| Saint Helena | N | Jamestown | 2 | 2 | 4 | 1 | 15 | 25 | 60 |
| Senegal | Y | Dakar | 7 690 | 8 605 | 16 294 | 2 978 | 18 | 29 | 53 |
| Sierra Leone | Y | Freetown | 3 247 | 4 473 | 7 720 | 1 136 | 15 | 27 | 58 |
| Togo | Y | Lomé | 3 332 | 4 659 | 7 991 | 1 746 | 22 | 20 | 58 |
| **TOTAL (West Africa)** |  |  | **177 189** | **204 792** | **381 981** | **39 191** | **10** | **36** | **54** |

Sources: United Nations, Department of Economic and Social Affairs, Population Division (2018). World Urbanization Prospects: The 2018 Revision, custom data acquired via website.

**CHILD MORTALITY**

**Fig. 10**: Median and interquartile range in under-five mortality rate (U5MR) by place of residence and region in sub-Saharan Africa (latest DHS or MICS survey, five-year rates)

**Table 3** Median and interquartile range (IQR) in under-five mortality rate (U5MR) by place of residence and region in sub-Saharan Africa (latest DHS or MICS survey, five-year rates, survey years 2010-2020)

| Median and IQR of U5MR | | | | | | |
| --- | --- | --- | --- | --- | --- | --- |
| Region | Capital |  | Other urban |  | Rural |  |
|  | Median | IQR | Median | IQR | Median | IQR |
| Central | 60.0 | 86.9 | 66.9 | 80.5 | 81.6 | 105.4 |
| Eastern | 57.2 | 67.0 | 51.6 | 58.7 | 62.1 | 51.5 |
| West | 57.6 | 73.5 | 75.7 | 84.4 | 105.7 | 113.6 |
| Southern | 63.4 | 68.9 | 57.7 | 36.1 | 66.8 | 20.2 |
| Total | 59.5 | 101.4 | 61.7 | 99.6 | 69.0 | 134.8 |

**Fig. 11**: Median and interquartile range in neonatal mortality rate (NMR) by place of residence and region in sub-Saharan Africa (latest DHS or MICS survey, five-year rates, survey period 2010-2020)

**Table 4** Median and interquartile range (IQR) in neonatal mortality rate (U5MR) by place of residence and region in sub-Saharan Africa (latest DHS or MICS survey, five-year rates, survey years 2010-2020)

| Median and IQR of U5MR | | | | | | |
| --- | --- | --- | --- | --- | --- | --- |
| Region | Capital |  | Other urban |  | Rural |  |
|  | Median | IQR | Median | IQR | Median | IQR |
| Central | 25.5 | 30.7 | 20.9 | 23.5 | 25.7 | 23.2 |
| Eastern | 27.3 | 39.9 | 25.8 | 21.0 | 26.1 | 9.2 |
| West | 20.5 | 35.2 | 29.2 | 27.3 | 29.2 | 23.0 |
| Southern | 24.7 | 22.6 | 23.0 | 21.3 | 29.6 | 13.6 |
| Total | 25.1 | 49.1 | 25.7 | 33.6 | 26.5 | 30.9 |

**Fig. 12** Trends in Under-five mortality by residence, Sub-Saharan Africa

**Table 5** Predicted slope of decline in under-five mortality by region

|  | | | Slope | | p-value | | 95%CI | |
| --- | --- | --- | --- | --- | --- | --- | --- | --- |
| Capital | | | -2.9 | | <0.001 | | (-3.4, -2.5) | |
| Other urban | | | -3.4 | | <0.001 | | (-3.8, -3.0) | |
| Rural | | | -4.1 | | <0.001 | | (-4.6, -3.7) | |
| CENTRAL AFRICA | | Slope | | p-value | | 95%CI | |  |
| Capital | | -3.5 | | <0.001 | | (-4.4, -2.7) | |  |
| Other urban | | -3.5 | | <0.001 | | (-4.2, -2.8) | |  |
| Rural | | -3.5 | | <0.001 | | (-4.6, -2.4) | |  |
| EASTERN AFRICA | | Slope | | p-value | | 95%CI | |  |
| Capital | | -3.5 | | <0.001 | | (-4.6, -2.5) | |  |
| Other urban | | -4.1 | | <0.001 | | (-4.7, -3.4) | |  |
| Rural | | -4.9 | | <0.001 | | (-5.7, -4.2) | |  |
|  | |  | |  | |  | |  |
| WEST AFRICA | | Slope | | p-value | | 95%CI | |  |
| Capital | | -3.0 | | <0.001 | | (-3.8, -2.5) | |  |
| Other urban | | -3.4 | | <0.001 | | (-4.1, -2.8) | |  |
| Rural | | -4.4 | | <0.001 | | (-5.2, -3.7) | |  |
|  |  |  | |  | |  | |  |
| SOUTHERN AFRICA | Period | Slope | | p-value | | 95%CI | |  |
| Capital | 2000-2004 | 1.4 | | <0.380 | | (-1.7, -4.4) | |  |
|  | 2004-2015 | -3.3 | | <0.194 | | (-1.7,4.4) | |  |
| Other urban | 2000-2004 | 0.8 | | <0.473 | | (-1.4,2.9) | |  |
|  | 2004-2015 | -4.5 | | <0.013 | | (-8.1,-1.0) | |  |
| Rural | 2000-2004 | 0.6 | | <0.461 | | (-0.9, 2.1) | |  |
|  | 2004-2015 | -3.6 | | <0.005 | | (-6.1,-1.1) | |  |

**Table 6** Predicted absolute gap in under-five mortality by residence in 1995, 2005, and 2015

|  | Absolute gap in U5MR | | | |
| --- | --- | --- | --- | --- |
|  | 2000 | 2005 | 2010 | 2015 |
| All sub-Saharan African | | | | |
| Other urban - Capital | 15 | 12 | 10 | 7 |
| Rural - Other urban | 26 | 23 | 19 | 15 |
| Rural - Capital | 41 | 35 | 29 | 23 |
| Central Africa | | | | |
| Other urban - Capital | 10 | 10 | 10 | 10 |
| Rural - Other urban | 21 | 21 | 21 | 21 |
| Rural - Capital | 31 | 31 | 31 | 31 |
| Eastern Africa | | | | |
| Other urban - Capital | 15 | 12 | 10 | 7 |
| Rural - Other urban | 20 | 16 | 12 | 7 |
| Rural - Capital | 35 | 28 | 21 | 14 |
| Southern Africa | | | | |
| Other urban - Capital | 8.3 | 4.1 | -4.7 | -13.6 |
| Rural - Other urban | 2.5 | 2.4 | 5.9 | 9.4 |
| Rural - Capital | 10.8 | 6.5 | 1.2 | -4.2 |
| West Africa | | | | |
| Other urban - Capital | 18 | 16 | 14 | 12 |
| Rural - Other urban | 39 | 34 | 29 | 24 |
| Rural - Capital | 57 | 50 | 43 | 36 |

**Table 7** Predicted under-five mortality rates and 95% confidence intervals by place of residence

| **Year** | **U5MR - Capital** | | | **U5MR - Other urban** | | | **U5MR - Rural** | | |
| --- | --- | --- | --- | --- | --- | --- | --- | --- | --- |
|  |  | 95%CI | |  | 95%CI | |  | 95%CI | |
|  | Estimates | UB | LB | Estimates | UB | LB | Estimates | UB | LB |
| PREDICTED MORTALITY RATES - ALL SSA | | | | | | | | | |
| 2000 | 100.5 | 109.4 | 91.6 | 115.4 | 124.1 | 106.6 | 141.4 | 153.2 | 129.7 |
| 2005 | 85.8 | 94.2 | 77.4 | 98.2 | 106.6 | 89.8 | 120.7 | 132.1 | 109.3 |
| 2010 | 71.1 | 79.7 | 62.4 | 81.0 | 89.5 | 72.5 | 100.0 | 111.5 | 88.5 |
| 2015 | 56.4 | 65.8 | 46.9 | 63.8 | 73.0 | 54.7 | 79.3 | 91.4 | 67.2 |
|  |  |  |  |  |  |  |  |  |  |
| PREDICTED U5MR - CENTRAL REGION | | | | | | | | | |
| 2000 | 109.0 | 86.4 | 131.7 | 118.6 | 97.8 | 139.3 | 139.5 | 113.3 | 165.8 |
| 2005 | 91.4 | 69.5 | 113.3 | 101.1 | 81.0 | 121.3 | 122.0 | 96.8 | 147.2 |
| 2010 | 73.8 | 51.9 | 95.7 | 83.7 | 63.5 | 103.9 | 104.4 | 79.2 | 129.6 |
| 2015 | 56.2 | 33.4 | 78.9 | 66.3 | 45.5 | 87.1 | 86.8 | 60.5 | 113.2 |
|  |  |  |  |  |  |  |  |  |  |
| PREDICTED U5MR - EASTERN REGION | | | | | | | | | |
| 2000 | 98.4 | 83.9 | 112.8 | 113.3 | 99.2 | 127.4 | 133.5 | 120.7 | 146.2 |
| 2005 | 80.7 | 66.6 | 94.7 | 92.9 | 79.0 | 106.9 | 108.8 | 96.2 | 121.3 |
| 2010 | 63.0 | 47.6 | 78.4 | 72.6 | 58.1 | 87.0 | 84.1 | 70.8 | 97.4 |
| 2015 | 45.3 | 27.1 | 63.5 | 52.2 | 36.6 | 67.8 | 59.4 | 44.5 | 74.3 |
|  |  |  |  |  |  |  |  |  |  |
| PREDICTION U5MR - SOUTHERN AFRICA | | | | | | | | | |
| 2000 | 82.66 | 59.01 | 106.31 | 90.93 | 70.55 | 111.31 | 93.44 | 80.70 | 106.18 |
| 2005 | 86.17 | 60.22 | 112.11 | 90.32 | 68.60 | 112.04 | 92.69 | 78.85 | 106.53 |
| 2010 | 76.35 | 53.94 | 98.76 | 71.62 | 52.00 | 91.24 | 77.52 | 65.34 | 89.70 |
| 2015 | 66.53 | 40.37 | 92.69 | 52.92 | 31.11 | 74.73 | 62.35 | 48.35 | 76.35 |
|  |  |  |  |  |  |  |  |  |  |
| PREDICTION U5MR - WEST AFRICA | | | | | | | | | |
| 2000 | 105.1 | 92.7 | 117.4 | 123.2 | 110.5 | 136.0 | 162.0 | 142.9 | 181.1 |
| 2005 | 90.0 | 78.2 | 101.7 | 106.0 | 94.1 | 117.9 | 139.9 | 121.5 | 158.2 |
| 2010 | 74.8 | 63.1 | 86.6 | 88.8 | 76.8 | 100.7 | 117.7 | 99.3 | 136.1 |
| 2015 | 59.7 | 47.3 | 72.2 | 71.5 | 58.6 | 84.4 | 95.6 | 76.4 | 114.7 |

**COVERAGE**

**Fig. 13** Median composite coverage index (CCI) by residence and sub-region in sub-Saharan Africa

**Table 8** Median composite coverage index (CCI) by residence and sub-region in sub-Saharan Africa

| Region | Capital | Other urban | Rural |
| --- | --- | --- | --- |
|  |  |  |  |
| Central | 62% | 54% | 41% |
| East | 73% | 72% | 66% |
| Southern | 76% | 78% | 74% |
| West | 63% | 60% | 50% |
|  |  |  |  |
| Total | 68% | 66% | 55% |

**Fig. 14** Trends in the composite coverage index (CCI) by residence

**Table 9** Slope and p-value of trends in CCI by place of residence

| Slopes |
| --- |
| Capital: 0.004, p<0.001 |
| Other urban: 0.005, p<0.001 |
| Rural: 0.010, p<0.001 |

**Table 10** Predicted average trends in CCI by place of residence and sub-region in sub-Saharan Africa

| Year | Capital cities | Other urban | Rural |
| --- | --- | --- | --- |
| **ALL** | | | |
| 2000 | 61% | 56% | 39% |
| 2005 | 64% | 59% | 44% |
| 2015 | 68% | 64% | 54% |
|  |  |  |  |
| **CENTRAL** | | | |
| 2000 | 56% | 48% | 33% |
| 2005 | 57% | 50% | 36% |
| 2015 | 61% | 54% | 40% |
|  |  |  |  |
| **EAST** | | | |
| 2000 | 65% | 61% | 42% |
| 2005 | 68% | 64% | 49% |
| 2015 | 74% | 70% | 61% |
|  |  |  |  |
| **SOUTHERN** | | | |
| 2000 | 75% | 74% | 63% |
| 2005 | 75% | 76% | 67% |
| 2015 | 75% | 76% | 68% |
|  |  |  |  |
| **WEST** | | | |
| 2000 | 59% | 52% | 33% |
| 2005 | 61% | 55% | 38% |
| 2015 | 64% | 60% | 48% |

**Table 11** Absolute gaps in CCI coverage

| Gap analysis | 2000 | 2005 | 2015 |
| --- | --- | --- | --- |
| Absolute gap Capital-Other urban | 5% | 5% | 4% |
| Absolute gap other urban - rural | 17% | 15% | 10% |
| Absolute gap capital - rural | 22% | 19% | 14% |

**DOUBLE DISAGGREGATION: PLACE OF RESIDENCE BY WEALTH QUINTILE**

**Fig. 15** Median and interquartile range for the composite coverage index for richest urban, poorest urban and rural by sub-region in sub-Saharan Africa

**Table 11** Median and interquartile range for the composite coverage index for richest urban, poorest urban and rural by sub-region in sub-Saharan Africa

| Region | Richest Urban | Poorest urban | Rural |
| --- | --- | --- | --- |
|  |  |  |  |
| Central | 65% | 40% | 43% |
| East | 74% | 62% | 66% |
| West | 67% | 50% | 48% |
| Southern | 79% | 80% | 73% |
| All | 69% | 55% | 51% |

**Fig. 16** Linear Prediction Double Disaggregation

**Table 12** Slope and p-value of trends in CCI by place of residence (linear prediction double disaggregation)

| Slopes |
| --- |
| Urban rich: .0045288, p<0.001 |
| Urban poor: .010572, p<0.001 |
| Rural: .0098307, p<0.001 |
